# Color of Copper/Copper Oxide

**DOI:** 10.1002/adma.202007345

**Published:** 2021-03-09

**Authors:** Su Jae Kim, Seonghoon Kim, Jegon Lee, Yongjae Jo, Yu‐Seong Seo, Myounghoon Lee, Yousil Lee, Chae Ryong Cho, Jong‐pil Kim, Miyeon Cheon, Jungseek Hwang, Yong In Kim, Young‐Hoon Kim, Young‐Min Kim, Aloysius Soon, Myunghwan Choi, Woo Seok Choi, Se‐Young Jeong, Young Hee Lee

**Affiliations:** ^1^ Crystal Bank Research Institute Pusan National University Busan 46241 Republic of Korea; ^2^ Research Institute of Basic Science Seoul National University Seoul 08826 Republic of Korea; ^3^ Department of Physics Sungkyunkwan University Suwon 16419 Republic of Korea; ^4^ Center for Neurscience Imaging Research Sungkyunkwan University Suwon 16419 Republic of Korea; ^5^ Department of Nanoenergy Engineering Pusan National University Busan 46241 Republic of Korea; ^6^ Division of High‐Tech Materials Research Busan Center Korea Basic Science Institute Busan 46742 Republic of Korea; ^7^ Center for Integrated Nanostructure Physics Institute for Basic Science Sungkyunkwan University Suwon 16419 Republic of Korea; ^8^ Department of Energy Science Sungkyunkwan University Suwon 16419 Republic of Korea; ^9^ Center for Artificial Synesthesia Materials Discovery and Department of Materials Science and Engineering Yonsei University Seoul 03722 Republic of Korea; ^10^ School of Biological Sciences Seoul National University Seoul 08826 Republic of Korea; ^11^ Department of Optics and Mechatronics Engineering Pusan National University Busan 46241 Republic of Korea

**Keywords:** atomic sputtering epitaxy (ASE), coherent oxidation, color control, interfaces, laser‐oxide lithography, single‐crystal copper thin films

## Abstract

Stochastic inhomogeneous oxidation is an inherent characteristic of copper (Cu), often hindering color tuning and bandgap engineering of oxides. Coherent control of the interface between metal and metal oxide remains unresolved. Coherent propagation of an oxidation front in single‐crystal Cu thin film is demonstrated to achieve a full‐color spectrum for Cu by precisely controlling its oxide‐layer thickness. Grain‐boundary‐free and atomically flat films prepared by atomic‐sputtering epitaxy allow tailoring of the oxide layer with an abrupt interface via heat treatment with a suppressed temperature gradient. Color tuning of nearly full‐color red/green/blue indices is realized by precise control of the oxide‐layer thickness; the samples cover ≈50.4% of the standard red/green/blue color space. The color of copper/copper oxide is realized by the reconstruction of the quantitative yield color from the oxide “pigment” (complex dielectric functions of Cu_2_O) and light‐layer interference (reflectance spectra obtained from the Fresnel equations) to produce structural color. Furthermore, laser‐oxide lithography is demonstrated with micrometer‐scale linewidth and depth through local phase transformation to oxides embedded in the metal, providing spacing necessary for semiconducting transport and optoelectronics functionality.

## Graphical abstract

Atomically flat perfect single‐crystal Cu films prepared by the atomic‐sputtering epitaxy method realize the coherent propagation of an oxidation front and display full‐color by controlling the oxide‐layer thickness. More than 50% of the standard red/green/blue color space is covered. Laser‐oxide lithography forming a microscale local oxide embedded in metal is introduced.

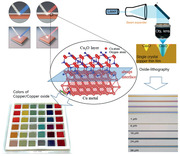

Surface oxidation of copper (Cu), one of the oldest problems in metallurgy, occurs naturally when Cu is exposed to air. The oxidation depends on the imposed environmental conditions.^[^ ^1^, ^2^, ^3^ ^]^ The tensor relationship between control parameters and oxidation has not been addressed thus far, because the propagation of oxidation occurs randomly at the surface with a high density of low coordinated surface sites (preferentially along grain boundaries). Oxidation is neither prevented nor systematically controlled along a uniaxial direction. Thus, systematic control over surface oxidation is necessary to take full advantage of the properties of metals.

Color modulation of metals has been attempted by exploitation of electrochromism, laser coloration using marking, piezochromism, and plasmonic effects.^[^ ^4^, ^5^, ^6^, ^7^ ^]^ Highly porous thin films on metal substrates with ultrathin, finely tuned optical coatings offer color purity enhancement.^[^ ^8^, ^9^, ^10^ ^]^ However, Cu and its alloys become tarnished and corrode under the ambient conditions often involved in antimicrobial applications on various touch surfaces in healthcare facilities.^[^ ^11^, ^12^ ^]^ Another strategy to obtain wide color selectivity in a metal film is the construction of sophisticated nanostructures to realize various colors by means of polarization conversion.^[^ ^13^ ^]^ Despite numerous attempts to modulate color by oxidation and nanostructuring efforts,^[^ ^14^, ^15^, ^16^ ^]^ the complexity associated with conversion of the Cu lattice into an oxide remains an obstacle for coherent control of the interface between metal and metal oxide, which is necessary to obtain a full, well‐defined color spectrum.

In this report, we present a breakthrough in the surface oxidation of Cu, using a grain‐boundary‐free, ultraflat single‐crystal Cu thin film (SCCF) prepared by atomic‐sputtering epitaxy (ASE). Inhomogeneous oxidation in the SCCF was highly suppressed by introduction of a treatment to minimize the temperature gradient in the film, resulting in the production of a full‐color spectrum by precise control of the oxide‐layer thickness. This approach is further extended to localized oxidation by laser‐oxide lithography for photonic–electronic applications.

**Figure** **1**a shows a schematic diagram of ASE, in which all internal electrical circuits are replaced by single‐crystal Cu wires instead of commercial Cu wires; the vibration due to ambient noise is highly suppressed by an anti‐vibration system (Experimental Section). However, ASE aims to realize atomically flat surfaces by stacking atom by atom. Hence, even minute vibration could significantly disturb initial nucleation and lateral growth, especially the coherent coplanar merging of the nuclei. We verified that precise signal transduction and cancellation of electrical interference through the use of grain‐boundary‐free wires were essential for acquisition of high‐quality Cu thin films. Indeed, ASE resulted in grain‐boundary‐free, SCCFs (Figure 1b top panel, SCCF) with a root mean square (RMS) roughness of 0.25 nm, comparable to the thickness of a single atomic layer along the (111) crystallographic direction of Cu (Figure S1, Supporting Information). To highlight the single‐crystal nature of our SCCF, we show the surface quality of a partially improved Cu thin film grown using single‐crystal target‐assisted sputtering (Figure 1b middle panel, intermediate),^[^ ^17^ ^]^ as well as a general polycrystalline Cu thin film (PCCF) grown using a conventional sputtering system [Figure 1b (bottom panel) and Figure S2, Supporting Information]. Numerous grains and grain boundaries were evident in scanning electron microscopy (SEM) images (left), with large RMS roughness values of 3.98 and 11.29 nm from atomic force microscopy (AFM) images (right); in contrast, the SCCF had an RMS roughness of 0.25 nm.

**Figure 1:**
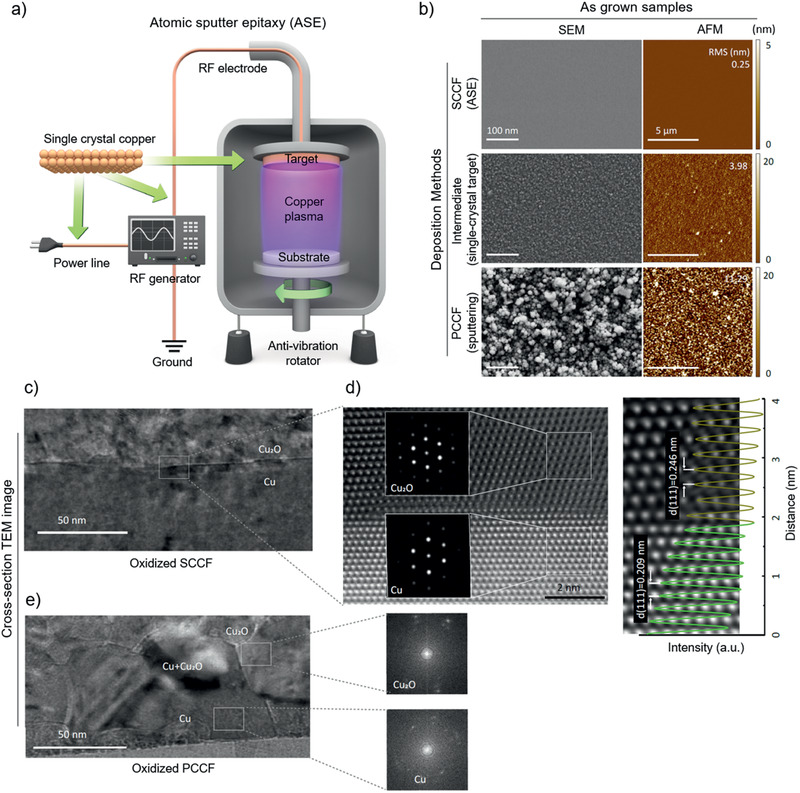
Improvement in copper (Cu) film crystallinity using ASE. a) Schematic diagram of ASE equipped with electrical single‐crystal Cu wiring and mechanical noise‐elimination system. b) SEM (left) and AFM (right) images of samples obtained from ASE, intermediate‐grade film using a single‐crystal Cu target, and a conventional sputtering system. c) Cross‐sectional TEM image of 50 nm SCCF (upper) after thermal treatment with an electron diffraction pattern near the interface in the inset. d) High‐resolution TEM image of marked area in (c) and a comparison of interplanar spacing profiles between Cu_2_O and Cu (right). e) TEM image of a PCCF with corresponding electron diffraction patterns.

A uniform, controllable oxide layer was obtained from the SCCF in a separate heating furnace. We annealed both SCCF and PCCF films at 330 °C for 1 min under a mixed‐gas atmosphere of Ar (83%) and O_2_ (17%). The SCCF exhibited an oxide layer with a highly crystalline Cu_2_O layer and nanometer‐scale abrupt interfaces. Figure 1 shows cross‐sectional high‐resolution (scanning) transmission electron microscopy ((S)TEM) images of the oxide layers of the SCCF (Figure 1c,d) and PCCF (Figure 1e); the interface between the crystalline Cu_2_O and SCCF layers was found to be atomically sharp (abrupt) showing the single‐crystallographic orientation by the fast Fourier transform (FFT) patterns (inset). Comparison of interplanar spacing profiles obtained along the out‐of‐plane direction of high‐angle annular dark‐field (HAADF)‐STEM images between Cu_2_O and the Cu film revealed that the Cu film possessed the layer spacing of the (111) stacking plane (dCu(111) = 0.21 nm); the oxidized area exhibited the larger lattice spacing of Cu_2_O (dCu_2_O(111) = 0.25 nm). In contrast, the conventional PCCF exhibited a rough surface and interface with blurry FFT patterns (Figure 1e). The interface misfit strain is steeply relaxed over about 2 nm or less by the presence of geometrical misfit dislocations and the residual strain is gradually relieved over about 4 nm into the Cu_2_O layer. (Figures S3 and S4, Supporting Information). We emphasize that coherent oxidation, with a highly crystalline Cu_2_O layer and nanometer‐scale abrupt interfaces, is critical for realization of homogeneous, genuine color in Cu films. This is achieved by minimization of the temperature gradient in the Cu film during heat treatment. For this purpose, we designed a double heating system equipped with an interior preheating furnace, in which the temperature was controlled to within ±0.1 °C (Experimental Section).

By dramatically improving the interface quality with controllable oxidation in the SCCF, we realized a wide‐color spectrum through exclusive use of simple heat treatment via temperature‐gradient minimization. **Figure** **2**a shows a photograph of representative SCCF samples with systematically controlled oxide‐layer thicknesses. This vivid representation of color, which was not previously achieved, emphasizes “color” as an indicator of the surface/interface quality of the Cu_2_O/Cu heterostructure; thus, it is an indicator of controlled oxidation. The color wheel (Figure 2b) shows representative colors of Cu films constructed from photographic images of actual samples (Figure S5, Supporting Information).^[^ ^18^, ^19^ ^]^ The range of colors achieved by our samples (>300) is mapped as a Commission Internationale de L'Eclairage *xy* chromaticity diagram (Figure 2c).^[^ ^20^ ^]^ Notably, colors from the SCCF cover ≈50.4% of the area of the standard red/green/blue (sRGB) color space of digitally available colors represented by the grey triangle, which represents enhancement of >250% in color coverage relative to a recent study regarding plasmonic color generation (18.4%).^[^ ^20^ ^]^ The photographic images were also obtained using near‐normal geometry. The spectra of incident light used for the images correspond to AM1.5. Note that the colors slightly change when different angle of incidence is used (Figure S6, Supporting Information).

**Figure 2:**
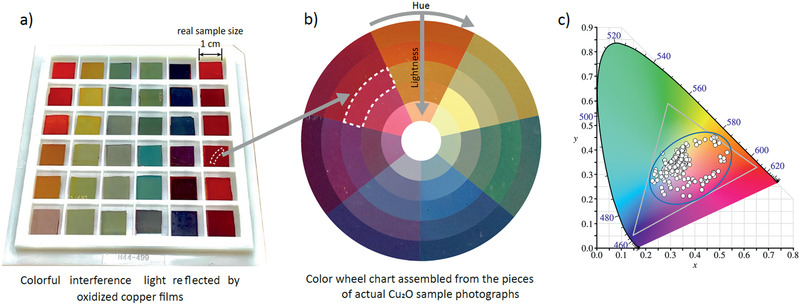
Photograph of color maps in SCCFs. a) Photograph of representative SCCFs. b) Color‐wheel photograph composed of the representative colors of actual samples. Samples are enumerated with hue in the clockwise direction of the wheel; lightness and saturation are demonstrated in the inward direction. c) Commission Internationale de L'Eclairage (CIE) xy chromaticity diagram with our samples (>300) and reference colors. The gray triangle represents sRGB color space of a typical computer monitor. The blue ellipse indicates range covered by the current work, as an area ratio relative to the gray triangle.

The underlying mechanism for the emergence of various colors can be explained in terms of the multiple reflections that occur at the oxidized film surface and interface between Cu_2_O and Cu,^[^ ^21^, ^22^ ^]^ as shown schematically in **Figure** **3**a. The reflected light depends strongly on the Cu_2_O thickness. The change in color of incident white light that occurs upon reflection, is determined by the dielectric functions of the material. First, the dielectric functions of our Cu_2_O layer are consistently obtained by spectroscopic ellipsometry measurements (Figures S7 and S8, Supporting Information).^[^ ^21^, ^22^, ^23^, ^24^, ^25^ ^]^ Based on the obtained dielectric functions of each layer, distinct reflectance spectra in the near‐normal incident geometry are simulated for specific CuO/Cu_2_O thicknesses, using the Fresnel equations. Figure 3b shows the simulated reflectance spectra (dotted lines. The simulated spectra are remarkably similar to experimental reflectance spectra (solid lines) of CuO/Cu_2_O/SCCF thin films annealed at 330 °C for various durations with Cu_2_O layer thicknesses of 0, 28, 35, and 60 nm, thus validating the thickness modulation of multiple reflections and realization of the color variation (Figure S9, Supporting Information).

**Figure 3:**
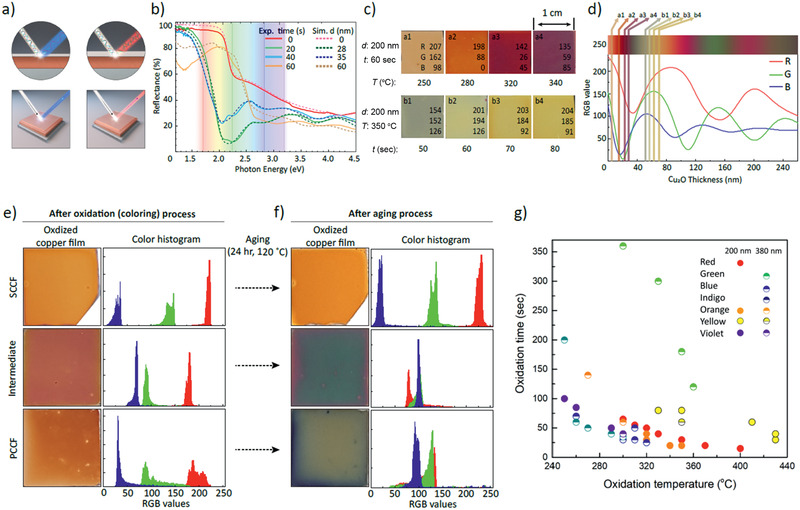
Reflectance spectra on Cu thin films. a) Schematic diagram of multiple reflections at optically abrupt interface of Cu_2_O/SCCF for thin (left) and thick (right) Cu_2_O layers. b) Reflectance spectra of SCCF annealed at 330 °C for annealing times of 0, 20, 40, and 60 s (solid lines) and simulated reflectance spectra extracted from the Fresnel equation, rationalizing the mechanism of color control of SCCF (dashed lines). c) (a1–a4) Variation of SCCF (*d* = 200 nm) by changing *T* from 250 to 340 °C for *t* = 60 s and (b1–b4) by changing t from 50 to 80 s at *T* = 350 °C with red/green/blue (RGB) digital color codes. d) Simulated reflectance spectra of RGB as a function of Cu_2_O‐layer thickness marked by layer thickness for (a1–a4) (between 5 and 30 nm) and for (b1–b4) (between 50 and 70 nm). e) Photographs of colored samples (left) and color histograms (right) after oxidation of samples shown in Figure 1b, f) After further aging for 24 h at 120 °C (of the samples shown in (e)). g) Seven representative colors with oxidation temperature (*T*) and duration time (*t*). Dots and half‐solid circles indicate respective thicknesses of 200 and 380 nm.

We investigated the critical variables for homogeneous color with a given initial Cu thickness (*d*) by controlling the annealing temperature (*T*) and annealing time (*t*). The systematic color variation from brown to purple was obtained with the corresponding RGB digital color codes by modulating *T* from 250 to 340 °C for *t* = 60 s and *d* = 200 nm (upper panel (a1–a4) in Figure 3c). This was equivalent to an oxide‐layer thickness range of 5 to 30 nm (Figure 3d). A different set of colors, from dark green to yellow‐brown, was modulated by adjusting *t* from 50 to 80 s at *T* = 350 °C for the same Cu thickness (*d* = 200 nm) (lower panel (b1–b4) in Figure 3c). This adjustment in the annealing time corresponds to an approximate copper oxide film thickness of 50 to 70 nm. RGB spectra were identified by simulating the reflectance spectra as a function of the Cu_2_O‐layer thickness for the given color samples, and by performing the reverse simulation (Figure 3d and Figure S10, Supporting Information). The abrupt change of thickness observed between a1‐4 and b1‐4 in Figure 3d occurred because the oxidation behavior changed abruptly at ≈350 °C, as shown by thermogravimetric analysis (Figure S11, Supporting Information).

The three samples in Figure 1b (specifically, the SCCF, intermediate film, and PCCF) exhibited the commonly formed brown color after oxidation at 260 °C for 1 min; the homogeneity, saturation, and lightness differed slightly among the film types (Figure 3e and Figure S12, Supporting Information). After further aging at 120 °C for 24 h in air, the color variation was remarkable (Figure 3f). A highly stable nature was retained in the SCCF, whereas the intermediate and PCCF samples were severely deteriorated. The color histogram in terms of RGB digital color codes (Figure 3e,f right panels) represents the tonal range from 0 (black) to 255 (white), as well as the number of pixels. The R, G, and B values in the color histogram are distinctly separate from each other, even in the aged SCCF sample. The observed broadening of distribution for the red values is attributed to a slight roughening of the surface or interface. (Figure S13, Supporting Information). In contrast, the intermediate and conventional PCCF samples after aging degraded to a highly inhomogeneous and darker achromatic color. Note that the initial Cu thickness also alters the color, although much less compared with the Cu_2_O thickness. Notably, the color change was almost negligible when the remaining Cu thickness exceeded ≈15 nm (Figure S9d, Supporting Information). Figure 3g shows seven representative colors enumerated in terms of their initial Cu thickness (200 to 380 nm); notably, precise control over the color with oxidation time and temperature is clearly demonstrated with a reproducibility of the mean variation of RGB values within ±3%. (Figure S14, Supporting Information).

We next investigated whether spatially confined control of oxidation of the SCCF could be realized using laser irradiation. A SCCF sample was mounted on a motorized stage and irradiated using a motorized shutter‐equipped continuous laser (wavelength: 488 nm). Notably, absorbance of the SCCF was ≈40% (Figure S15, Supporting Information). Reflectance was subsequently measured by a color scientific complimentary metal–oxide–semiconductor (sCMOS) camera. Precise control of both laser intensity and duration was required to balance photothermal heating and conductive cooling. The samples were irradiated with a focal spot of 2 µm in diameter (*e* ^−2^) at varying irradiance of 5 to 3500 kJ mm^−2^, by modulating the pulse number *N* (**Figure** **4**a). Immediately after irradiation, colorful multilayered concentric circles were visible in the SCCF, with diameters ranging from 1.7 to 4.5 µm. The circular color pattern exhibited a smooth transition from the center to the surrounding area, suggesting that the depth profile of oxidation was also gradual under irradiation of 2500 to 3500 kJ mm^−2^ on the SCCF; notably, only 5 kJ mm^−2^ was required for the PCCF. Importantly, the laser dose required to create an oxidation pattern of similar size in the SCCF was ≈80‐fold greater than that of the PCCF (Figure 4b). The heat‐affected zone in the PCCF was ≈tenfold larger than that of the SCCF for a similar size of oxidation pattern. These results can be explained by the degree of defects or grain boundaries, which allow for more rapid and longer propagation of oxidation in the PCCF, compared to the SCCF.

**Figure 4:**
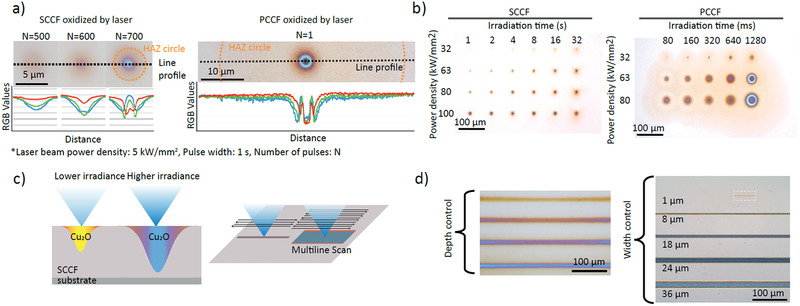
Laser‐oxide lithography and line profiles. a) Photographs and RGB‐color line profiles for focally irradiated SCCFs and PCCFs at varying optical fluence (laser beam power density: 5 kW mm^−2^, pulse width: 1 s, number of pulses: *N*). b) Focal oxidation of SCCF and PCCF samples with varying irradiation time and power density. c,d) schematic diagram and experimental result of optical patterning by laser‐oxide lithography with irradiance power. Focal oxidation (zero dimension, as a dot) can be concatenated to form lines (in one dimension) with depth control indicated by coloration, as well as rectangular patterns (two dimensions).

After establishing focal oxidation (0D) as a dot, we investigated whether line (1D) or area (2D) oxidation could be achieved. To deliver the controlled exposure in a predefined pattern, we scanned the sample stage in sync with the laser shutter. Figure 4c,d shows 1D controlled oxidation in the SCCF by varying the laser power (32–100 kW mm^−2^ with a fixed scanning speed of 200 m s^−1^ and a loop number, *N*, of 500). The AFM images (Figure S16, Supporting Information) shows the trace of the laser after irradiating 1 and 2.5 min with a fixed laser intensity of 100 kW mm^−2^_._ The longer irradiation time resulted in appreciable surface roughening due to lattice expansion during oxide formation. Using a raster scan, 2D patterns were also created, as shown in Figure 4d. The 1D oxidation lines evident in Figure 4d still have a color gradient, which depends on the minimum pixel size of laser‐induced lithography and limits potential applications. The color gradient can be improved by adopting top‐hat beam shapers.^[^ ^26^ ^]^ Collectively, our results show that arbitrary patterning of oxidation in the SCCF at near‐diffraction‐limited precision offers new opportunities for laser‐oxide lithography on metal films.

Our results demonstrate that oxidation of the SCCF can be controlled coherently with atomically flat precision. The thickness of the Cu_2_O layer on the SCCF was manipulated to within an accuracy of 2–3 nm, with a well‐defined interface between the oxide and the metal. The colors realized on the SCCF, as demonstrated in our findings, represent the coherence of oxidation front propagation in the Cu lattice, which is a good indicator of oxidation thickness. As an application of Cu oxidation to the photonic‐electronic area, we introduce a laser‐oxide lithography technique by local oxidation that can be tuned from 1 μm to several tens of micrometers in diameter for 0D, 1D, and 2D patterns.

## Experimental Section

### Preparation of Oxidized Cu Thin Film

ASE was adopted to grow a pristine SCCF, which is an improved method achieved by modifying the technical limits of a conventional sputtering system. ASE deposition enhances the quality of the metal film and supplies nearly defect‐free and grain‐boundary‐free SCCFs. The system was improved by minimization of signal noise originating from grain boundary scattering of electrons in conductors through replacement of the conventional configuration with a single‐crystal Cu wiring network.^[^ ^27^, ^28^, ^29^ ^]^ Mechanical noises from other equipment, including motors and pumps, also contribute to rough surfaces and defect formation in the films; thus, in the set‐up, any vibration caused by ambient noise was minimized to the fullest extent using an anti‐vibration system. An4ti‐vibration techniques during bulk single‐crystal growth have been previously introduced.^[^ ^30^, ^31^ ^]^ In general, lower vibration of the growth system should improve the film crystallinity during thin film growth as well. In reality, minute vibration does not yield noticeable degradation in conventional thin film growth, especially for the polycrystalline thin films. Meanwhile, ASE aimed to realize atomically flat surfaces by stacking atom by atom. Hence, even minute vibration could significantly disturb initial nucleation and lateral growth, especially coherent coplanar merging of nuclei. The ASE helps to provide perfect plasma gases during the sputtering process. Consequently, the Cu film prepared by ASE showed improved RMS roughness, to ≈0.2 nm (2 Å) under optimal conditions and ≈0.3 nm on average (from ≈9.1 nm in a previous study).^[^ ^17^ ^]^

Oxidation of the Cu film using the aforementioned SCCF was controlled by the following parameters: treatment temperature, treatment time, oxygen partial pressure, and pre‐treatment Cu film thickness. The most important factor for homogeneous color is the ability to eliminate the temperature gradient in the sample during the oxidation process. In particular, a heating furnace was designed, which was equipped with a preheating chamber connected to a gas inlet. The temperature gradient in the preheating chamber was <0.1 °C at ≈300 °C. The sample was inserted into the heated chamber to instantaneously reach the target temperature. The sample was immediately quenched to room temperature after a specific time interval. The treatment temperature of the oxidation process for Cu_2_O formation varied from 230–430 °C, according to the target color. The treatment time was occasionally as short as 10 s; in most instances, it did not exceed 5 min. A thick CuO phase (thicker than native CuO layer of >3 nm, generally forms on the top of Cu_2_O layer) emerged when the samples were annealed >330 °C for >5 min. The samples were treated under a mixed‐gas atmosphere of Ar (83%):O_2_ (17%).

### Structural Characterization

X‐ray diffraction θ–2θ measurements were performed using a PANalytical Empyrean Series 2 system (Malvern Panalytical, Malvern, UK), equipped with a Cu‐Kα source (40 kV, 30 mA). Data were collected within the range of 20° < 2θ < 90°, with a step size of 0.0167° and a dwell time of 0.5 s per point in all instances. AFM measurements were carried out using an XE‐100 system (Park Systems, Inc., Suwon, Korea). SEM, electron backscatter diffraction, pole figure, and inverse pole figure measurements were performed with a Zeiss SUPRA40 VP (Carl Zeiss AG, Oberkochen, Germany), using a scanning electron microprobe. High‐resolution TEM analyses were performed using an FEI Titan 3 G2 60–300 (FEI/Thermo Fisher Scientific, Waltham, MA, USA), equipped with double aberration correctors (image and probe) and a monochromator operating at an acceleration voltage of 200 kV. TEM samples were prepared with a focused ion beam (Helios 450F1; FEI/Thermo Fisher Scientific).

### Optical Characterization

Optical reflectance measurements were conducted at room temperature using a near‐infrared–visible–ultraviolet spectrometer (Lambda 950; Perkin Elmer Inc., Waltham, MA, USA) over the spectral range of 200–2000 nm. Reflectance measurements were carried out at near‐normal incidence geometry (≈10°). A flat aluminum mirror was used as a reference. Optical dielectric functions were characterized using spectroscopic ellipsometry. Ellipsometry spectra were obtained using a rotating‐compensator ellipsometer (M‐2000; J.A. Woollam, Co., Lincoln, NE, USA) and a rotating‐polarizer ellipsometer (VVASE; J.A. Woollam, Co.) at room temperature. A photon energy range of 0.74–5.5 eV was employed, with incident angles of 60°, 70°, and 80°. To obtain the layer structure and dielectric functions of oxidized Cu thin films, optical analyses were performed step‐by‐step using WVASE software. Figure S7, Supporting Information, shows the experimental spectroscopic ellipsometry data and standard fitting procedure. In all procedures, *Ψ*(ω) and Δ(ω) were fitted, which are the ellipsometric angles obtained from the intensity and phase ratio of the reflectance for s‐ and p‐ polarized light, respectively, to minimize the mean squared error (MSE). A lower value of MSE means that the fitted spectra are closer to the experimental spectra, indicating higher fitting quality. The authors began with the known reference complex (real and imaginary) dielectric functions (ε_1_(ω) and ε_2_(ω)) of Cu_2_O and Cu and fitted the thickness of the Cu_2_O layer (Figure S7a, Supporting Information); the initial dielectric functions of the layers were adapted from Palik.^[^ ^32^ ^]^ A thin CuO layer and surface roughness layer were added to improved further the fitting (Figure S7b,c, Supporting Information), respectively. The layer structure was thus surface roughness/CuO/Cu_2_O/Cu. Finally, all parameters were optimized together to obtain the result and the actual complex dielectric functions of Cu_2_O. The final fitted results of the different samples are shown in Figure S8, Supporting Information. Consistent fitting of *Ψ*(ω) and Δ(ω) for the different samples provided reliable ε_1_(ω) and ε_2_(ω) values for Cu_2_O (Figure S8d, Supporting Information). Furthermore, the reflectance spectra of oxidized Cu thin films were simulated using the above model layer structure. Change in the reflectance spectra was intuitively understood from the oscillation of color shown in Figure S9d,e, Supporting Information, which are reflectance spectra simulations of the sample geometries using the Fresnel equations shown in the inset. The actual fitting of optical functions was necessary only for the Cu_2_O layer, due to its dominant thickness effect on the optical response of the overall heterostructure. The CuO thickness influenced the color, but the influence was nearly negligible because the native CuO layer was thin without much modulation in thickness. Ellipsometry fitting provided the native CuO thickness of 3 ± 0.5 nm for the best fit. This is negligible compared with the controlled Cu_2_O thickness of 28.3–59.9 nm established by optical analyses (Figures S7 and S8, Supporting Information).

### Setup of Laser‐Oxide Lithography and Characterization

To build up oxide lithography on the SCCF, the following parts were assembled into a microscope. A continuous laser (LBX‐488‐500, Oxxius, Lannion, France) with 500 mW power at 488 nm was used to heat the SCCF, given that Cu has a relatively high absorbance below wavelengths of 532 nm. A motorized shutter (SH05, Thorlabs, Newton, NJ, USA) was controlled using software provided by the manufacturer. A 10× objective lens (numerical aperture: 0.45; CFI Plan Apo lambda, Nikon Corp., Tokyo, Japan), a 50:50 beam splitter (BS013, Thorlabs), and a tube lens (TTL200, Thorlabs) for a sCMOS camera (Kiralux CS895CU, Thorlabs) were also included in the lithography system. To oxidize the metal film using the laser, a 2‐µm‐diameter (1/*e* ^2^) laser beam was focused onto the SCCF and the power density was varied (200, 400, 800, and 1600 W mm^−2^) with 100 ms of irradiation. Notably, 100 W mm^−2^ irradiation was attempted, but clear pattern generation could not be achieved. In addition, laser irradiation with a power density >1600 W mm^−2^ produced irregular circular shapes, possibly indicating melting of the metal. A supercontinuum broadband laser beam (EXW‐12, NKT Photonics, GmbH, Cologne, Germany) was focused on the pattern with a 1‐µm^2^ spot size. The reflected signal was relayed to a spectrophotometer (Semrock SR‐303i Spectrograph and Newton DU970‐bv EMCCD, Andor/Oxford Instruments). Scanning of the laser pattern was performed using a motorized microscope stage (Ti2‐e, Nikon Corp.), controlled by software (NIS‐Elements, Nikon Corp.).

## Conflict of Interest

The authors declare no conflict of interest.

## Supporting information

Supporting Information

## Data Availability

Research data are not shared.
