# Supplementary material for: Color of Copper/Copper Oxide
Source: Adv Mater. 2021 Mar 9;33(15):2007345. doi: 10.1002/adma.202007345 (PMC11469196; doi:10.1002/adma.202007345)
Supplement: Supplementary file 1 — Supporting Information [file ADMA-33-2007345-s001.pdf]

© 2021 Wiley-VCH GmbH

ADVANCED MATERIALS

Supporting Information

for *Adv. Mater.,* DOI: 10.1002/adma.202007345

Color of Copper/Copper Oxide

Su Jae Kim, Seonghoon Kim, Jegon Lee, Yongjae Jo, Yu-Seong Seo, Myounghoon Lee, Yousil Lee, Chae Ryong Cho, Jong-pil Kim, Miyeon Cheon, Jungseek Hwang, Yong In Kim, Young-Hoon Kim, Young-Min Kim, Aloysius Soon, Myunghwan Choi, Woo Seok Choi, Se-Young Jeong,* and Young Hee Lee**

Supporting Information

Color of Copper/Copper Oxide

Su Jae Kim[†], Seonghoon Kim [†], Jegon Lee[†], Yongjae Jo, Yu-Seong Seo, Myounghoon Lee, Yousil Lee, Chae Ryong Cho, Jong-pil Kim, Miyeon Cheon, Jungseek Hwang, Yong In Kim, Young-Hoon Kim, Young-Min Kim, Aloysius Soon, Myunghwan Choi, Woo Seok Choi[*], Se-Young Jeong[*], & Young Hee Lee[*]

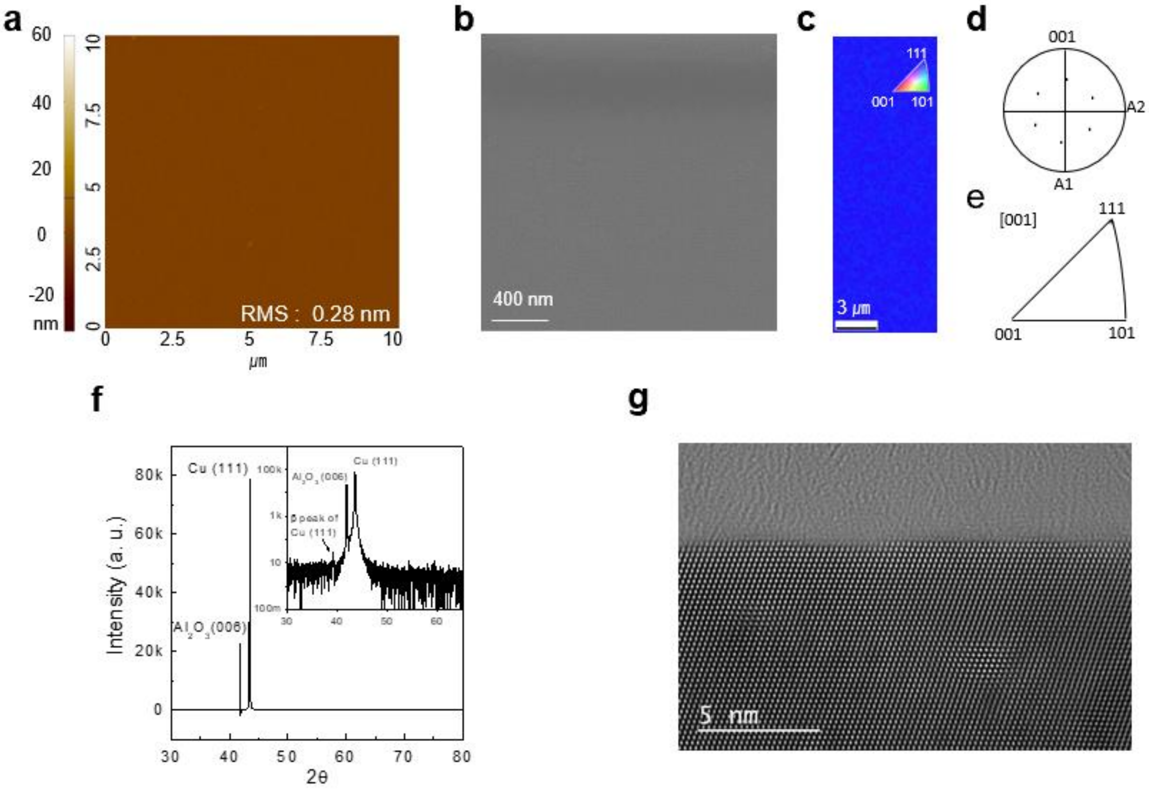

Figure S1. Pristine SCCF before heat treatment. a) Surface morphology from atomic force microscopy (AFM) images, with a root-mean-square (RMS) surface roughness of 0.25 nm. b) Scanning electron microscopy (SEM) image exhibiting no grain boundaries at the 400-nm scale. c) Electron backscatter diffraction mapping showing perfect alignment along the (111) plane. d) [100] pole figure showing six-fold symmetry of the {100} plane. e) Inverse pole figure with a sole spot associated with the (111) plane. f) $\theta-2\theta$ X-ray diffraction (XRD) data with a full width at half maximum of 0.060°, comparable to that of Al_2O_3 (0.048°). g) Surface of Cu film grown along the [111] direction, with a monolayer-thick step-edge structure observed using cross-sectional (scanning) TEM with 5-nm resolution.

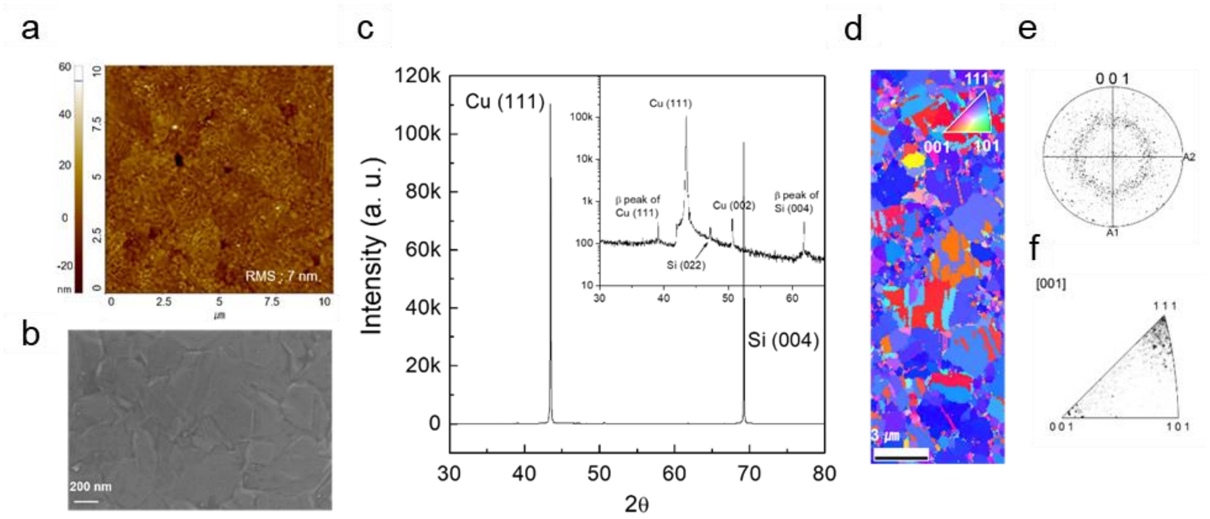

Figure S2. PCCF before thermal treatment. a) Surface morphologies from AFM images with a surface RMS roughness of 7 nm. b) SEM image showing grain boundaries of ~10^9/cm^2. c) θ−2θ XRD data exhibiting mixed phases of (111) and (200). d) Electron backscatter diffraction map depicting numerous grains with different crystallographic orientations. e) [100] pole figure showing various, but less obvious, speckles. f) Inverse pole figure revealing a blurred (111) plane and additional spots.

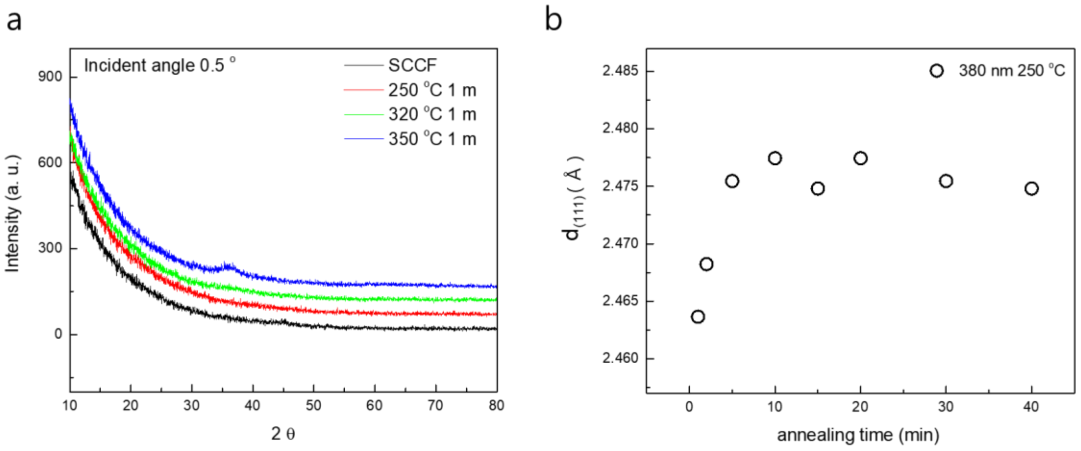

Figure S3. a) Grazing incident X-ray diffraction (GIXD) results of films treated for 1 min at different temperatures. b) Change of $d_{Cu2O(111)}$ of the samples treated at 250 °C for different annealing times.

Figure S3a shows the GIXD results at the incident angle of 0.5º for the SCCF annealed for 1 min at 250 °C, 320 °C, and 350 °C. Only the sample annealed at 350 °C showed a small peak near 36º, which reflects that the sample treated at 350 °C has a relatively strained region due to the nonlinear behavior of oxidation above ~ 350 °C. This result shows that that the Cu_2O phase in our color samples treated below 330 °C was uniformly aligned in the (111) direction and had crystallographically well ordered orientation.

Figure S3b shows the change of the Bragg peak position of the sample treated at 250 °C as a function of annealing time to investigate the lattice expansion according to the sample treatment conditions. While 1-minute-annealed sample showed relatively large change of lattice constant due to the influence of the rest Cu, the lattice constant was stabilized with increasing annealing time (increasing Cu_2O thickness). Samples treated longer than 2 min, where the thickness of Cu_2O exceeded ~15 nm, appeared to be relaxed without strain.

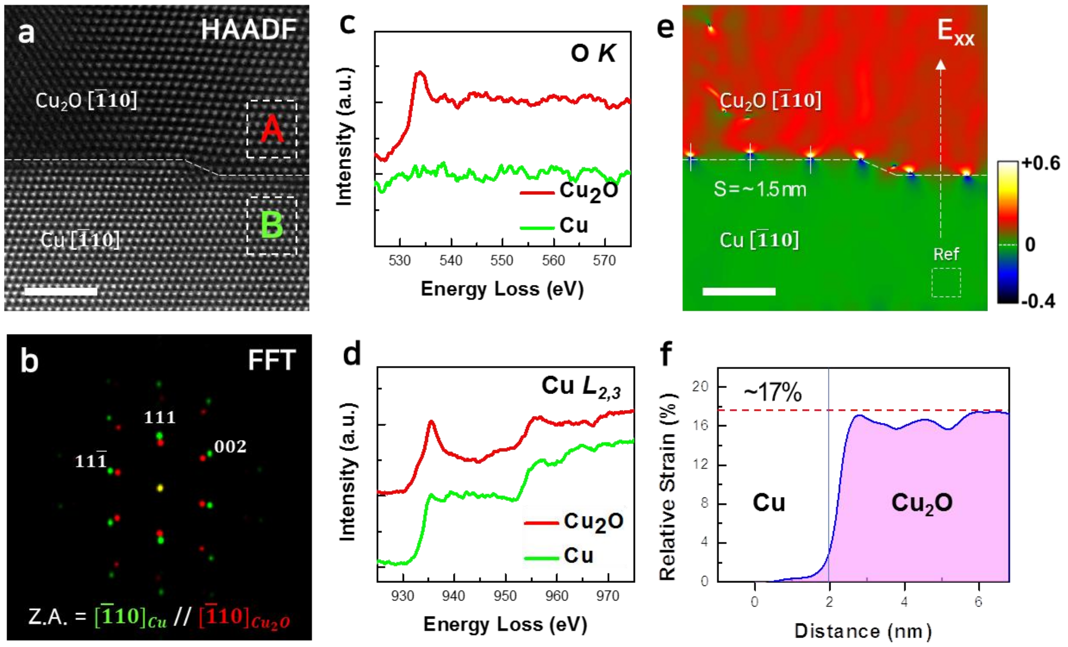

Figure S4. Strain distribution across the interface between Cu_2O and Cu. a) Cross-sectional high-angle annular dark-field scanning transmission electron microscope (HAADF STEM) image of the interface of the Cu_2O/Cu heterostructure. b) Fast Fourier transform (FFT) pattern of the HAADF STEM image showing the crystallographic orientation relationship of the two crystals as $(111)_{Cu_2O}[\bar{1}10]_{Cu_2O}$ //$(111)_{Cu}[\bar{1}10]_{Cu}$. c,d) Electron energy loss spectra of O *K* and Cu $L_{2,3}$ edges obtained from the Cu_2O (marked by A, red) and Cu (marked by B, green) layers, respectively. e) In-plane lattice strain map (E_{xx}) obtained by geometrical phase analysis (GPA) of the HAADF STEM image, in a). The color scale of the GPA map denotes magnitude of the strain relative to the reference Cu region marked by the white dotted box. f) Strain profile relative to the Cu lattice across the interface obtained along the dotted arrow in e. Scale bars correspond to 2 nm.

Figure S4 shows the strain relaxation behavior of the oxide layer via geometrical phase analysis of the cross-sectional interface structure at the atomic scale. The interface is found to be atomically sharp (*abrupt*) and layer mismatch is noticeably observed at the step edge of the Cu surface. Considering the orientational relationship, the misfit strain (δ) was calculated as ~17%. In this highly lattice mismatched system, it is expected that the in-plane lattice strain is majorly released by repetitive introduction of geometrical misfit dislocation (MD) because lattice mismatch can be more efficiently accommodated by a combination of elastic strain and MDs rather than elastic strain alone. The theoretical average spacing (S) of MDs was estimated as ~1.5 nm according to the relationship of $S = (|b|\cos 30^{\circ})/\delta$, where |b| is the magnitude of the Burgers vector of the perfect dislocation. To understand the microscopic in-plane strain (E_{xx}) relaxation behavior of the grown Cu_2O film, we performed geometrical phase analysis (GPA) of the atomic-resolution STEM image.[*Ultramicroscopy* **74** (1998) 131–146] It is evident that the misfit dislocation cores are repetitively placed along the Cu_2O/Cu interface and the distance between them is in very good agreement with a predicted spacing of about 1.5 nm. This result clarifies that most of the misfit strain was relieved by the periodic array of the interface misfit dislocations. The interface misfit strain is steeply relaxed over about 2 nm or less by the presence of MDs and the residual strain is gradually relieved over about 4 nm into the Cu_2O layer. (Figure S4f)

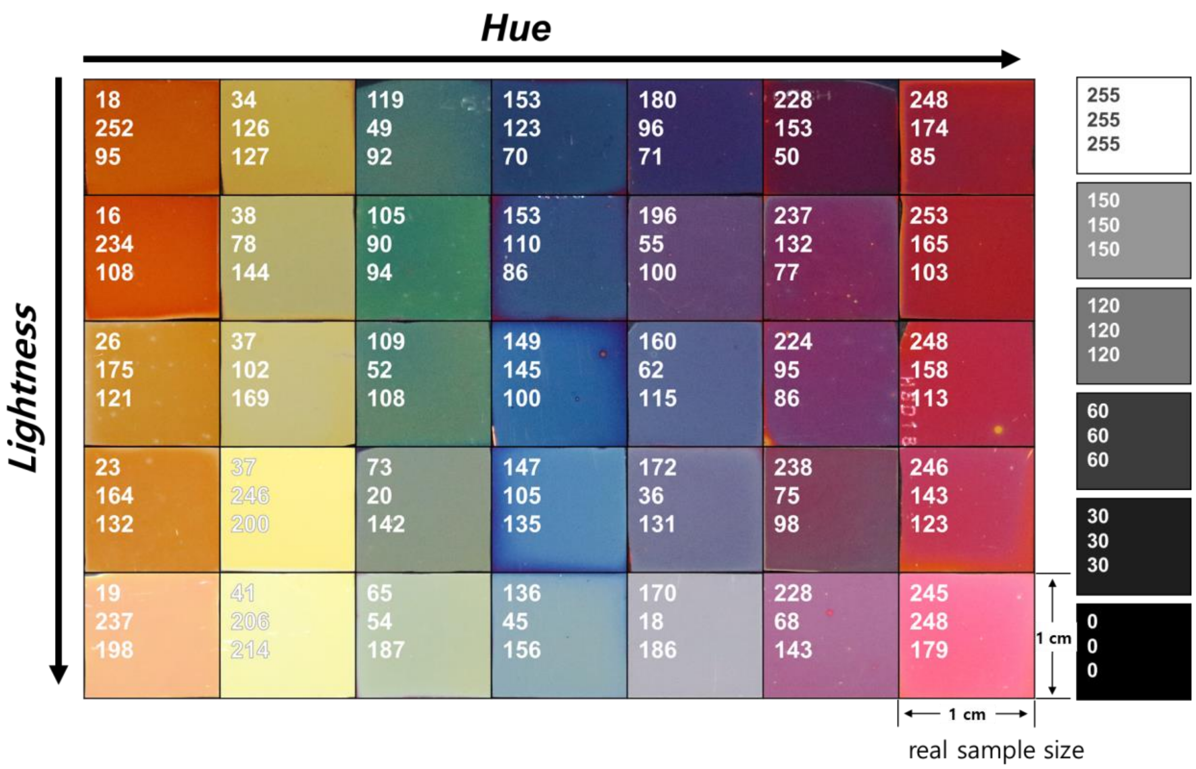

Figure S5. Colors implemented on the surface of the Cu thin film. The 35 samples shown in the color wheel of Figure 2b are enumerated along the *x*-axis with increasing hue, and along the *y*-axis with increasing saturation and lightness. Numbers on each sample indicate hue, saturation, and lightness (HSL). For convenience, the HSL ranges (0–255, 0–255, 0–255) follow the standard given in Microsoft PowerPoint. The samples obtained in this study demonstrate different HSL values; however, we selected 35 samples that belonged to a specific color space for better readability. By controlling the parameters of treatment temperature, atmosphere, treatment time, and pristine Cu thickness, we were able to obtain >350 colors. The achromatic colors on the right are provided as visual absolute references.

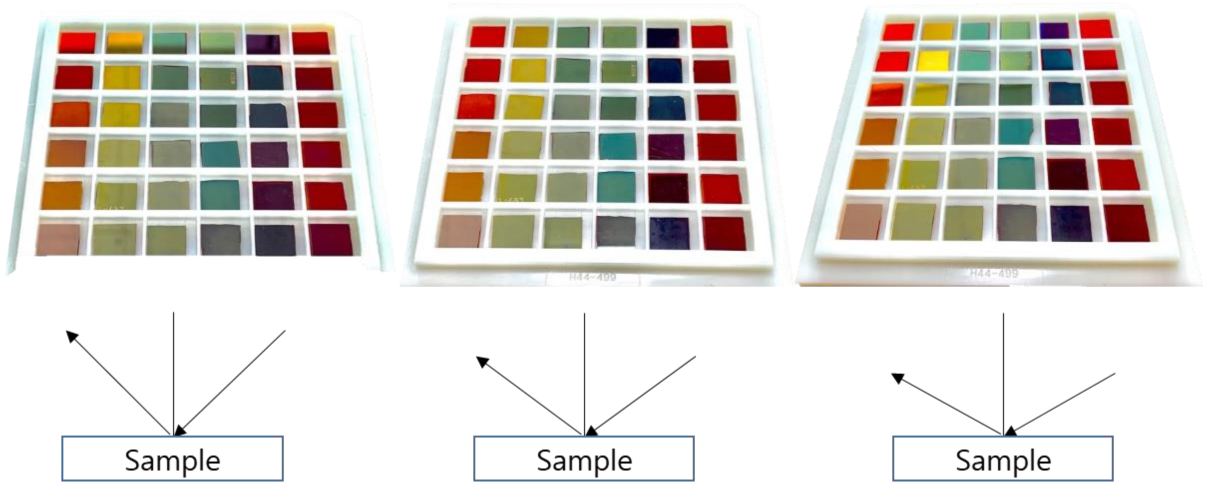

Figure S6. Incident angle-dependent color change. As the incident angle changes, the Fresnel equations yield different spectrum of the reflected light, although the color change is minimal for the angles used here.

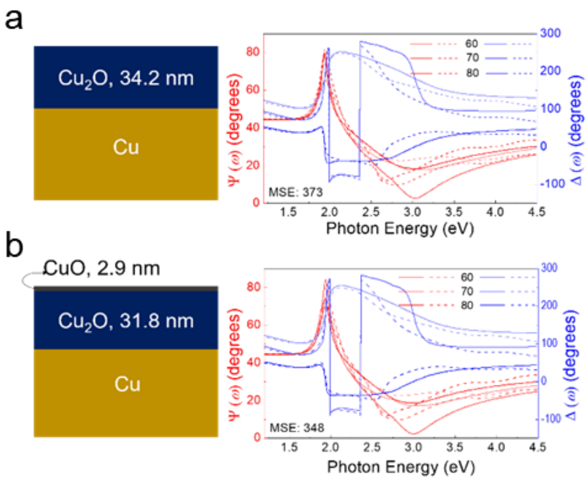

Figure S7. Fitting of ellipsometry data. Right panels show comparison between experimental and fitted $\Psi(\omega$, red), and $\Delta(\omega$, blue) spectra for ellipsometry measurements performed at 60°, 70°, and 80°. Straight and dotted lines correspond to fitted and measured data, respectively. Fitting steps are as follows. a) Fitting of Cu_2O-layer thickness. b) Simultaneous fitting of thicknesses of CuO and Cu_2O layers. c) Adding surface roughness layer (3 nm) with obtained thicknesses of CuO and Cu_2O layers. Thus far, optical functions of Cu, Cu_2O, and CuO are from the literature.[16,23] d) Fitting of optical functions of Cu_2O layer. During this procedure, the mean-square-error (MSE) was consistently reduced, from 373, 348, and 320 to 53.

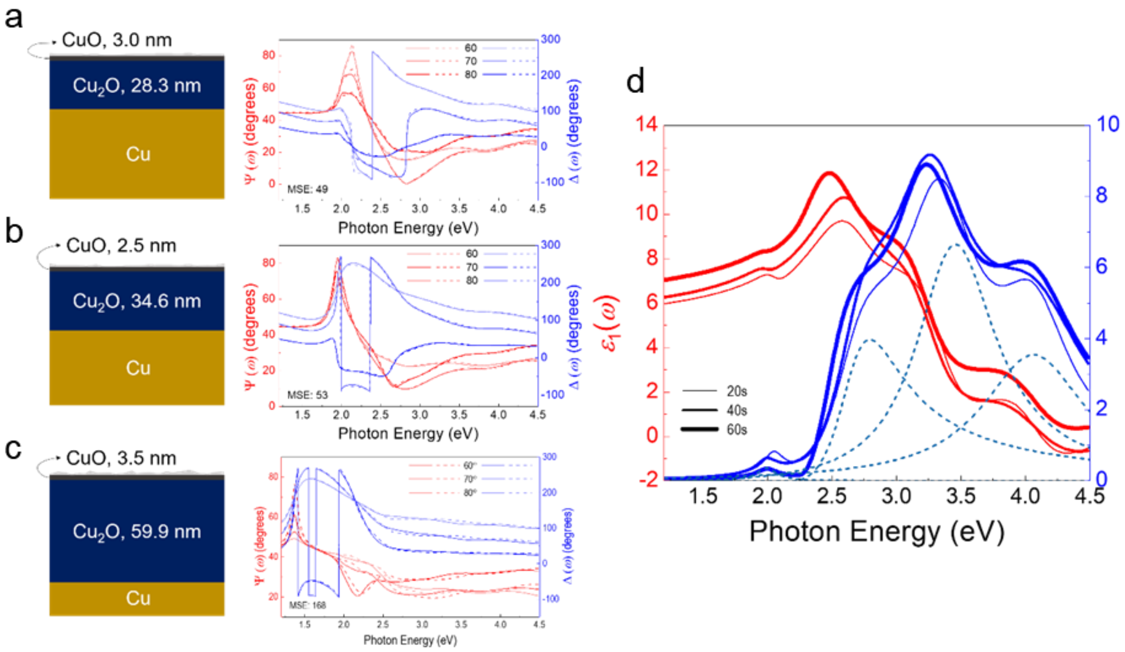

Figure S8. Final fitting result of ellipsometry data of CuO/Cu2O/Cu heterostructures shown in main figure. a), b), and c) Right panels show comparison between experimental and fitted $\Psi(\omega$, red) and $\Delta(\omega$, blue) spectra for ellipsometry measurements performed at 60°, 70°, and 80°. Straight and dotted lines are fitted and measured data, respectively. Optical properties of SCCFs annealed at 360 °C for 20 s (a), 40 s (b), and 60 s (c) are shown. MSEs of each fitting are 49, 53, and 349, respectively. d) Real [$\varepsilon_1(\omega)$, red] and imaginary [$\varepsilon_2(\omega)$, blue] parts of optical functions of Cu2O layer obtained from spectroscopic ellipsometry measurements. Dotted lines present four Lorentz and Tauc–Lorentz oscillators corresponding to particular optical transitions. Each oscillator represents optical transitions of electrons from valence to conduction state, creating electronic structure of Cu2O.

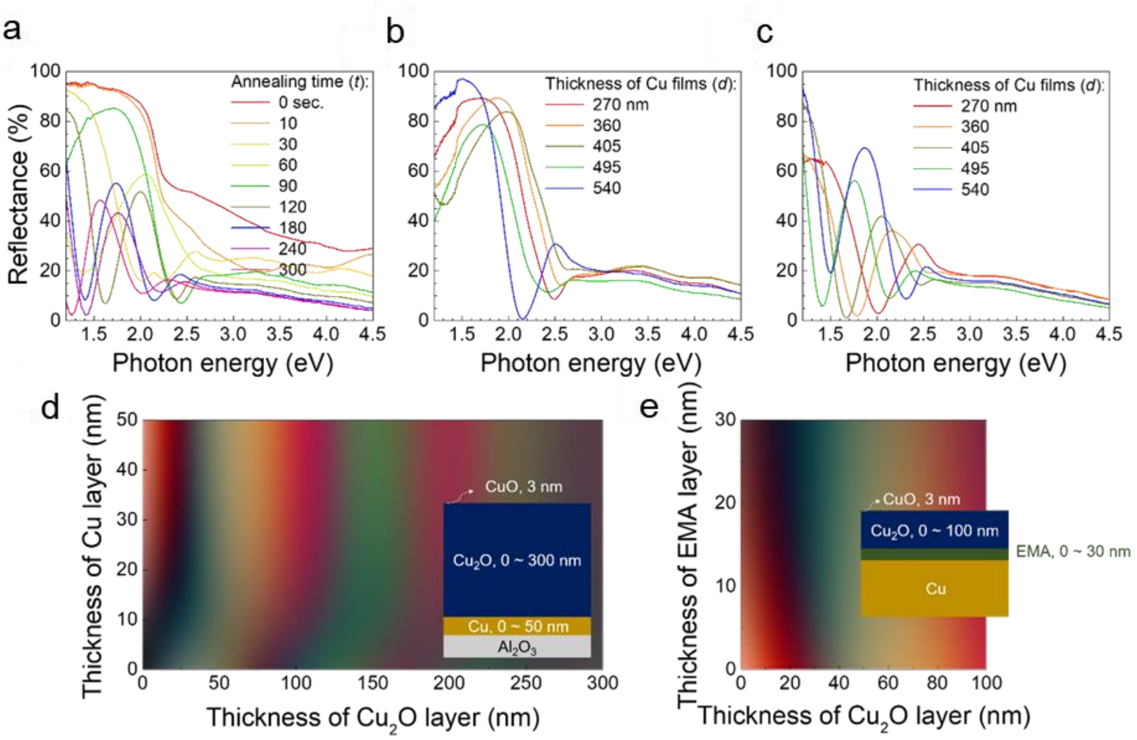

Figure S9. Reflectance spectra of oxidized Cu thin films and possible color palette of simulated layer structures. a) *t*-dependent reflectance spectra with $T = 330$ °C and $d = 405$ nm. b) and c) *d*-dependent reflectance spectra with $T = 350$°C and $t = 1$ min (b) and 2 min (c). d) and e) Simulated color of [$CuO/Cu_2O/Cu/Al_2O_3$] (d) and [CuO/Cu_2O/EMA/Cu] layer structures (e) of various thicknesses, respectively (simulation performed using WVASE software). In the simulation, intermediate layer was constructed using an effective medium approximation (EMA) between dielectric functions of Cu and Cu_2O layers; optical functions of each layer were taken from *Palik's Handbook of Optical Constants of Solids*.

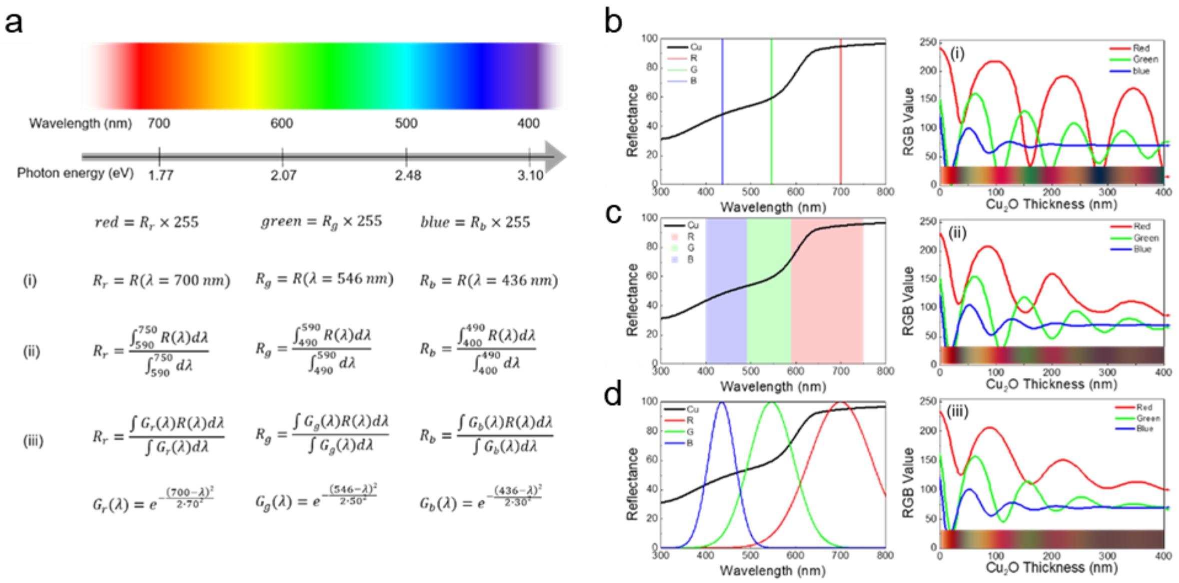

Figure S10. Translation of reflectance spectra to RGB color code. a) Analogue color can be simply translated into digital color code using three methods, based on $R(\lambda)$ (i.e., reflectivity as a function of wavelength). (i) Values of RGB color codes calculated by reflectivity at 700, 546, and 436 nm, representing red, green, and blue, respectively. (ii) Values of RGB color codes calculated by normalizing reflectivity across a certain wavelength range. Wavelength regions of 590–750, 490–590, and 400–490 nm were used to represent red, green, and blue, respectively. (iii) Values of RGB color codes calculated by normalizing weighted reflectivity using a Gaussian function. Gaussian functions centered at 700, 546, and 436 nm were used to represent red, green, and blue, respectively. b), c), and d) Each schematic diagram (left panels) and RGB color result (right panels) correspond to methods shown in panels (b), (c), and (d). RGB color result is obtained by simulating reflectance spectra of heterostructure $CuO/Cu_2O/Cu$, with systematic changes in Cu_2O-layer thickness. A variety of colors can be realized by changing thickness, indicating effectiveness of oxidation technique. Each color code shows oscillating behavior with different oscillation periods determined by frequency of light.

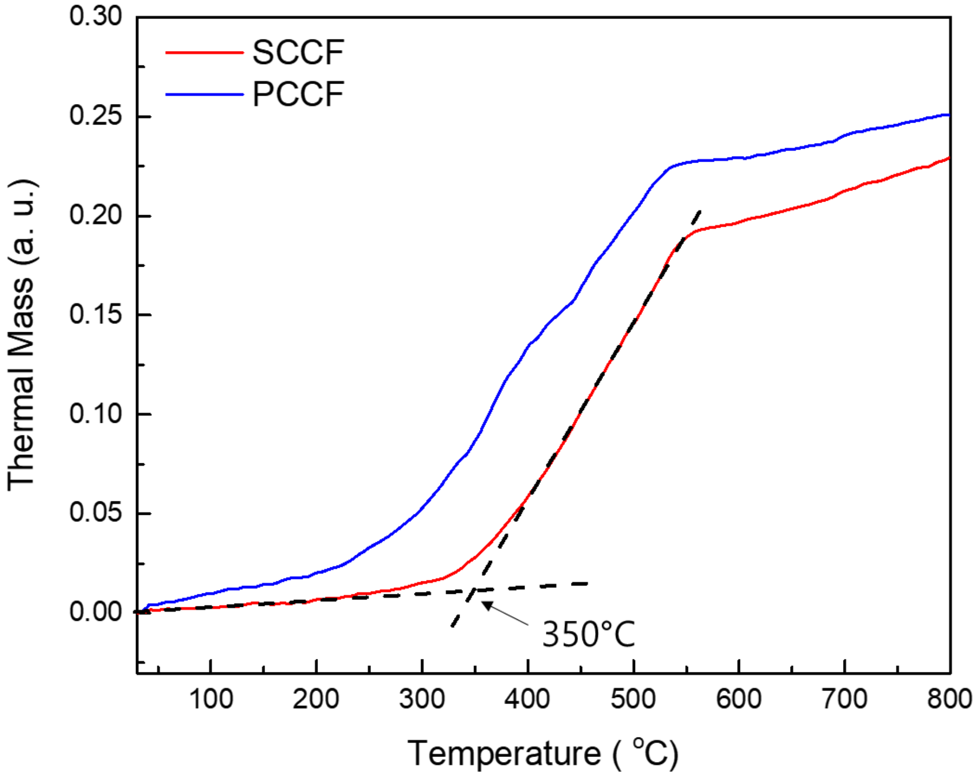

Figure S11. Thermogravimetric-Analysis (TGA) result that measured with heating rate 20 °C/min. (SCCF: single-crystal copper thin film, PCCF: polycrystalline copper thin film)

The temperature dependent oxidation behavior of a Cu thin film is rather different from that of the bulk crystal or polycrystalline thin film. Thermogravimetric-analysis (TGA) measured at the heating rate of 20 °C/min (Figure S11) shows that the SCCF thermal mass abruptly at ~350 °C due to oxidation, whereas the PCCF thermal mass increased rather gradually from 220 °C. This result supports the idea that the oxidation behavior of SCCF changes abruptly at 350 °C, in which corresponds to the drastic change of Cu_2O thickness noted by the reviewer.

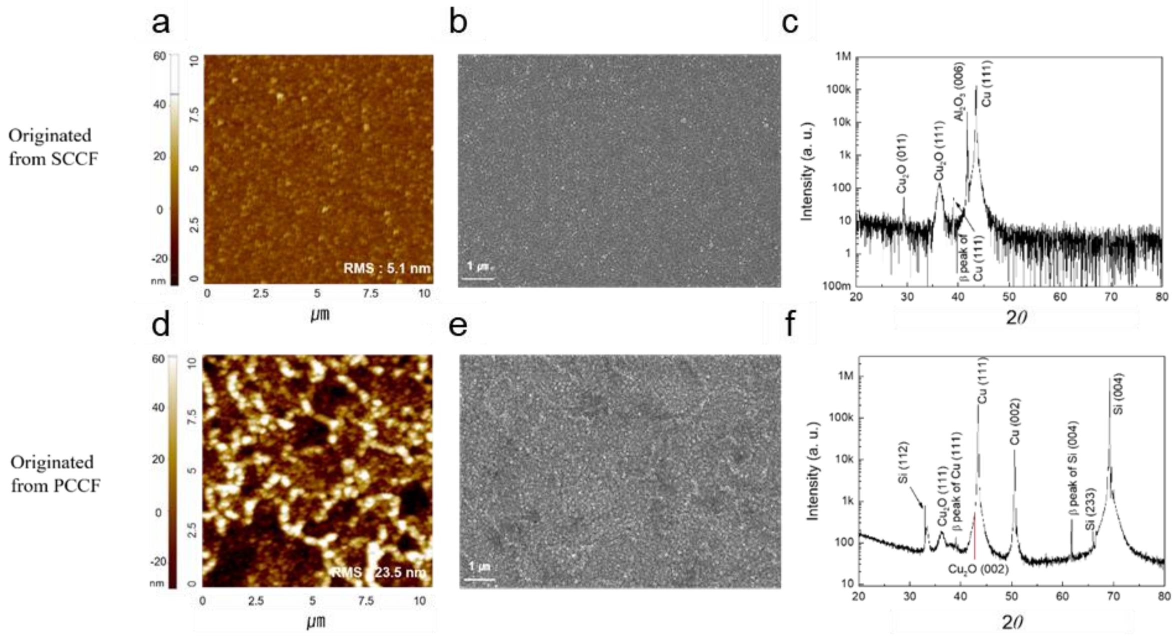

Figure S12. SCCF and PCCF after heat treatment. a) Surface morphology obtained from AFM images of SCCF after thermal treatment at 260 °C for 1.5 min; RMS surface roughness was 5.1 nm. b) SEM image of SCCF after thermal treatment. c) θ–2θ XRD data of SCCF after thermal treatment showing high crystallinity along the (111) direction. d) Surface morphology from an AFM image of PCCF after thermal treatment at 260 °C for 1.5 min; RMS surface roughness was 23.5 nm. e) SEM image of PCCF after heat treatment. f) θ–2θ XRD data of PCCF after thermal treatment showing mixed Cu_2O(111) and Cu_2O(200) phases. Figure S12 shows that heat-treated sample from SCCF had epitaxially grown a Cu_2O phase, while the remaining Cu (111) remained unchanged, creating an abrupt interface between oxide and metal where incident light was reflected. However, PCCF with a mixed phase of Cu(111) and Cu(200) was transformed into a mixed phase film of Cu_2O(111), Cu_2O(200), Cu(111), and Cu(200) after heat treatment, where incident light was scattered irregularly.

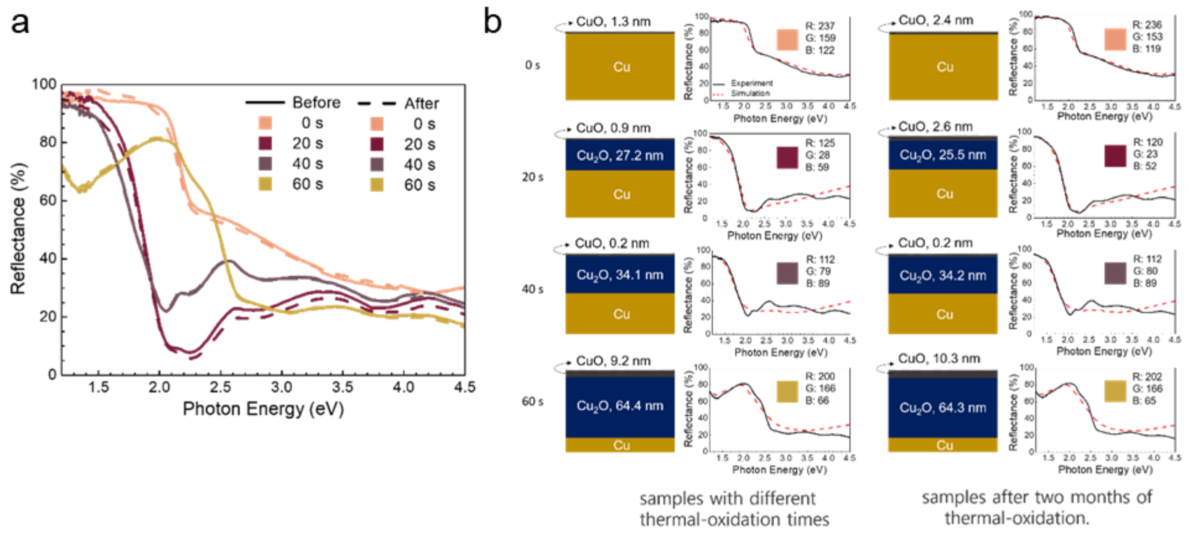

Figure S13. Stability of oxidization layer and color of Cu. a) Change in reflectance spectra of Figure 3b over time. Even after 2 months, change in color was minimal. b) Reflectance spectrum fitting indicates that oxidation layer does not become thicker over time under ambient conditions. Fitting was carried out using library Cu_2O, with a surface roughness of 1.5 nm.

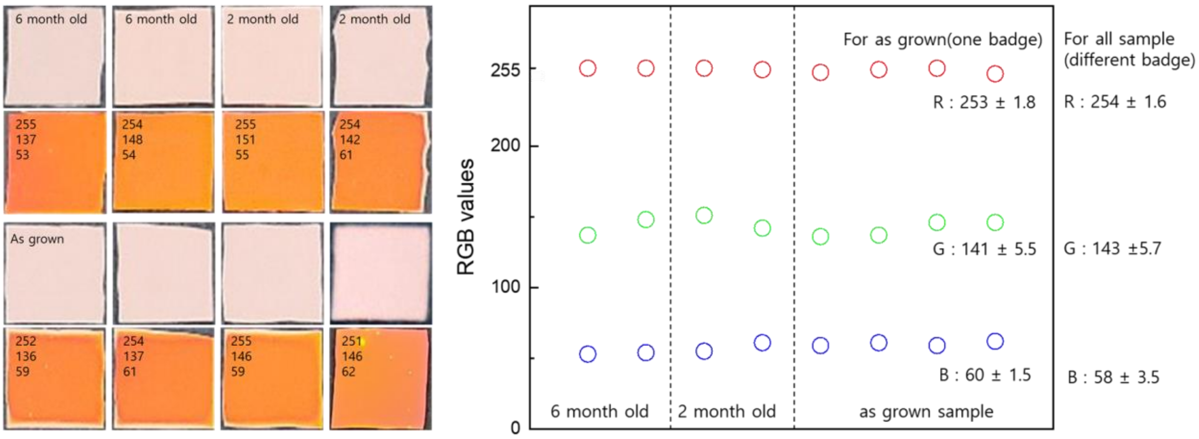

Figure S14. Reproducibility of the color from SCCF. The SCCF sample that heat treated at 250 ℃, for 2 min for as grown, 2 month old, 6 month old samples.

Coloration of the SCCF was relatively reproducible. Control of the oxide layer thickness was carried out with an accuracy of 2–3 nm, and this tight control resulted in good color reproducibility. Sometimes, depending on the degree of surface roughness of the initial Cu samples, a slight color variation was evident. However, using the same oxidation temperature and time resulted in the same color within the mean variation of the RGB values of ~ 2.8% (± 3.6/255) and the colors obtained from high-quality Cu films displayed consistent color within ~ 2.3% (± 2.9/255). The colors obtained from 2- and 6-month-old and as-grown SCCF samples are presented in Figure S14. While the value of R was relatively invariant, those of G and B changed slightly more than red.

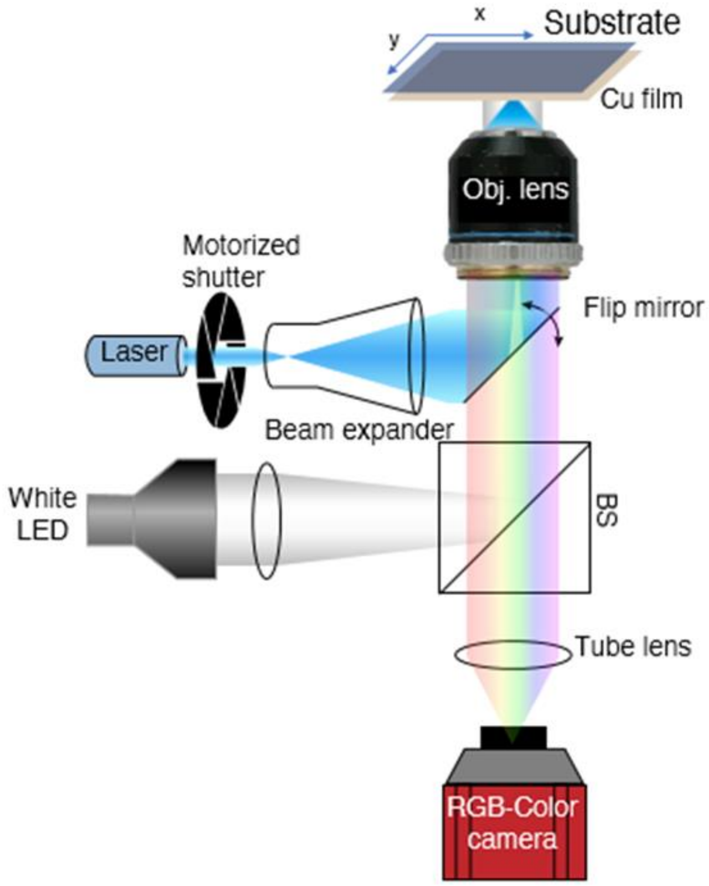

Figure S15. Schematic images of sample mounting stage for oxide lithography using a laser. Optical setup for laser irradiation and colorimetric analysis. Cu film is irradiated by a focused 488-nm laser and observed by a color sCMOS camera. BS, beamsplitter; sCMOS, scientific complimentary metal-oxide-semiconductor.

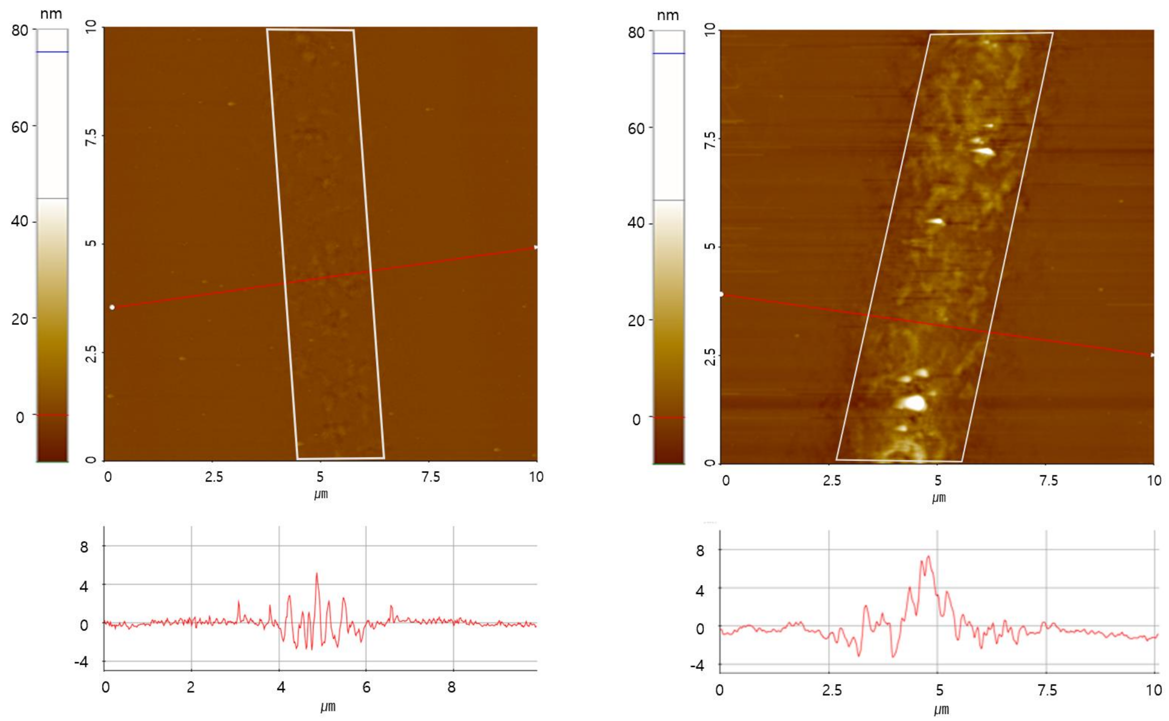

Figure S16. AFM image of the colored area after laser irradiation for a) 1 min and b) 2.5 min. The areas marked by white box.

The lattice constant of Cu, $d_{Cu(111)}$ and Cu_2O, $d_{Cu2O(111)}$ along the [111] direction is 3.615 Å and 4.26 Å, respectively. The 1.17-times larger lattice constant of Cu_2O means that lattice expansion by oxidation is inevitable.

After irradiating with a laser intensity of 100 kW mm^{-2} for 1 min during a fixed-loop scan (200 m s^{-1} speed, 2 Hz repetition rate), the surface morphology was not appreciably altered. The treated area is marked by a white box in Figure S16a. The line profile below the image indicates a few nanometers of thickening after irradiation. The surface morphology change became more apparent after increasing the irradiation time to 2.5 min. We also observed that the surface height increased with increasing irradiation time (Figure S16b) but the width of the irradiance trace was well maintained. This result supports the idea that coherent oxidation propagation in the depth direction of an SCCF can be achieved by precise control of various laser irradiance parameters, such as intensity, pulse width, repetition rate, treatment duration, wavelength, and beam profile.
